# Comparison of Prostate Cancer Detection Between an MRI-Directed Fusion Biopsy Strategy and Conventional Systematic Biopsy: A Retrospective Cohort Study

**DOI:** 10.3390/cancers18121936

**Published:** 2026-06-14

**Authors:** Chih-Wei Wu, Yu-Cheng Lu, Chen-Hsun Ho, Thomas I-Sheng Hwang, Te-Fu Tsai, Chung-Hsin Yeh, Guang-Dar Juang, Yi-Hong Cheng, Kuang-Yu Chou, Hung-En Chen, Chu-Tung Lin, Ping-Jui Lee, Allen W. Chiu, Chao-Yen Ho

**Affiliations:** 1 Division of Urology, Department of Surgery, Shin Kong Wu Ho-Su Memorial Hospital, Taipei 11101, Taiwan; jackmarswu@gmail.com (C.-W.W.); m016970@ms.skh.org.tw (Y.-C.L.); m015695@ms.skh.org.tw (C.-H.H.); m001009@ms.skh.org.tw (T.I.-S.H.); m004400@ms.skh.org.tw (T.-F.T.); m000732@ms.skh.org.tw (C.-H.Y.); m000733@ms.skh.org.tw (G.-D.J.); m002067@ms.skh.org.tw (Y.-H.C.); ky_chou@livemail.tw (K.-Y.C.); m002020@ms.skh.org.tw (H.-E.C.); m015908@ms.skh.org.tw (C.-T.L.); m016520@ms.skh.org.tw (P.-J.L.); 2 School of Medicine, College of Medicine, Fu Jen Catholic University, New Taipei City 242062, Taiwan; 3 School of Medicine, National Yang Ming Chiao Tung University, Taipei 30010, Taiwan

**Keywords:** MRI-directed fusion biopsy, systematic biopsy, prostate cancer

## Abstract

**Background/Objectives**: Prostate cancer (PCa) detection via a conventional systematic biopsy (SB) may miss cancer lesions, promoting the exploration of alternative methods. Multiparametric MRI demonstrates high sensitivity and specificity for the detection of clinically significant prostate cancer and an MRI-directed three-dimensional fusion biopsy (MFB) strategy may improve cancer detection rates. There are limited data available in Taiwan regarding this novel technique. This study aimed to compare the accuracy of cancer detection rates between MFB and SB. **Methods**: From January, 2021 through October, 2023, patients with PSA levels of 4–20 ng/mL and no palpable prostate nodules were retrospectively reviewed. They were categorized into the MFB and SB groups. Clinical parameters and PCa detection rates were compared between the two groups. Multivariable logistic regression analyses were performed to identify independent predictors of cancer detection. A separate subgroup analysis was conducted within the MFB cohort to evaluate the association between the PI-RADS category and biopsy outcomes. **Results**: A total of 262 patients (89 in MFB and 173 in SB) were included in the final analysis. MFB demonstrated significantly a higher detection rate in detecting PCa compared to SB (45.0% vs. 31.8%, *p* = 0.036). Furthermore, MFB exhibited superior rates in detecting clinically significant PCa (csPCa) and high-grade PCa compared to SB (40.4% vs. 22.5%, *p* = 0.002; 20.2% vs. 5.8%, *p* ≤ 0.001, respectively). The MRI PIRADS score exhibited a positive correlation with the detection of PCa, csPCa, and high-grade PCa (*p* ≤ 0.001, *p* ≤ 0.001, and *p* = 0.015, respectively). After an adjustment for age, PSA level, and PSAD, MFB remained independently associated with overall PCa detection (adjusted OR 2.01, 95% CI 1.16–3.48, *p* = 0.013), csPCa detection (adjusted OR 2.83, 95% CI 1.58–5.08, *p* < 0.001), and high-grade PCa detection (adjusted OR 6.15, 95% CI 2.49–15.19, *p* < 0.001). PSAD was also independently associated with all cancer outcomes. Within the MFB cohort, PI-RADS 5 lesions demonstrated significantly higher odds of overall PCa (adjusted OR 7.38, 95% CI 2.18–24.98, *p* = 0.001), csPCa (adjusted OR 8.42, 95% CI 2.49–28.49, *p* < 0.001), and high-grade PCa detection (adjusted OR 6.93, 95% CI 1.82–26.41, *p* = 0.005) compared with PI-RADS 3 lesions. **Conclusions**: In this retrospective cohort, the MFB strategy was associated with higher detection rates of PCa, csPCa, and high-grade PCa compared with conventional SB. PSAD and MRI findings independently contributed to cancer prediction, supporting the integration of clinical and imaging parameters in prostate biopsy decision-making. These findings support the clinical value of MRI-directed biopsy strategies but should be interpreted cautiously because of the non-randomized allocation and the differences in biopsy route and sampling strategy.

## Simple Summary

Between January 2021 and October 2023, patients with PSA levels of 4–20 ng/mL and no palpable prostate nodules underwent either MFB or SB according to clinical decision-making and patient preference. A total of 262 patients were analyzed (89 MFB, 173 SB). MFB was associated with significantly higher overall PCa detection rates than SB (45.0% vs. 31.8%, *p* = 0.036). It also showed a superior detection of clinically significant PCa (40.4% vs. 22.5%, *p* = 0.002) and high-grade disease (20.2% vs. 5.8%, *p* < 0.001). Additionally, MRI PIRADS scores were positively correlated with the detection of PCa, clinically significant PCa, and high-grade PCa. Multivariable logistic regression analysis demonstrated that MFB remained independently associated with cancer detection after an adjustment for age, PSA level, and PSA density. In addition, within the MFB cohort, PI-RADS 5 lesions were independently associated with significantly higher odds of overall, clinically significant, and high-grade prostate cancer detection compared with PI-RADS 3 lesions. These findings indicate that MFB provides an improved cancer detection rate over SB and highlight the clinical value of MRI-based targeting in prostate biopsy strategies.

## 1. Introduction

Managing men with elevated prostate-specific antigen (PSA) levels poses a complex challenge for urologists. Conventionally, individuals with an elevated PSA level are recommended to undergo a transrectal ultrasonography-guided biopsy (TRUSP-Bx) of the prostate, involving 10 to 12 cores. However, TRUSP-Bx is blind to the location of cancer in the prostate, which leads to the overdetection of low-grade prostate cancers (PCa) and the underdetection of high-grade cancers [1]. TRUSP-Bx also carries significant morbidity, such as hematuria, hematospermia, rectal bleeding, or infection, and can cause life-threatening sepsis [2].

Implementing an alternative pathway that incorporates imaging for assessing men with an elevated PSA level could significantly enhance diagnostic precision while minimizing unnecessary biopsies. Recent EAU guidelines recommend an MRI assessment to guide the prostate biopsy in suspected prostate cancer [3]. Over the last decade, the efficacy of multi-parametric Magnetic Resonance Imaging (mpMRI) in identifying and excluding clinically significant PCa has notably advanced [1]. mpMRI excels in detecting higher-risk PCa while systematically disregarding low-risk disease, which makes it an appealing prospect as a potential screening tool [4]. Employing mpMRI as a preliminary test could potentially avoid the need for a biopsy in cases of negative results, while positive findings could facilitate a targeted examination of suspicious prostate lesions during the biopsy [5].

Large prospective studies support the diagnostic role of mpMRI before a prostate biopsy. In the PROMIS paired validating study, mpMRI showed a high sensitivity for clinically significant prostate cancer and allowed a substantial proportion of men to avoid an immediate biopsy while improving the detection of clinically significant disease. The PROMIS trial demonstrated a sensitivity of 93% and specificity of 41% for clinically significant prostate cancer detection using mpMRI, highlighting its value as a triage test before biopsy [1]. In the PRECISION randomized trial, an MRI-targeted pathway detected more clinically significant cancer than a standard biopsy (38% vs. 26%) and reduced the detection of clinically insignificant disease. A Cochrane review similarly reported the high sensitivity of MRI-based pathways for Gleason score ≥ 7 cancer, although the specificity was lower, indicating that MRI should be integrated with clinical risk factors rather than interpreted in isolation [1,6,7]. The initial development of elastic fusion systems aimed to enhance the accuracy of a prostate biopsy by aligning the prostate contour during imaging fusion [8]. Today, these fusion systems have gained widespread adoption globally. Currently, there are few imaging fusion systems commercially accessible for targeted prostate biopsy. Notably, in Taiwan, the introduction of fusion systems did not occur until 2020. Before this milestone, patients underwent fusion biopsy using the In-Bore technique, performed within the MRI scanner by radiologists. The procedure typically took 30 min for one target, with an additional 15 min for each subsequent one. In our study, our primary objective was to compare the prostate cancer detection rates between the MRI-directed fusion biopsy (MFB) strategy and conventional systematic biopsy (SB) strategy in patients with PSA levels between 4–20 ng/mL without palpable prostate nodules. The primary outcome measure was the overall prostate cancer detection rate, while the secondary outcomes included the clinically significant prostate cancer (csPCa) and high-grade prostate cancer detection rates. We hypothesized that an MRI-directed biopsy strategy would be associated with higher detection rates of clinically significant disease compared with the conventional systematic biopsy [9].

## 2. Materials and Methods

### 2.1. Participants and Study Design

This study was designed as a retrospective cohort study conducted at a single tertiary referral center. Between 1 January 2021 and 31 October 2023, a total of 283 patients exhibiting PSA levels within the range of 4–20 ng/mL and no palpable prostate nodules were initially recruited from Shin Kong Wu Ho-Su Memorial Hospital. The exclusion criteria included individuals with any of the following conditions: (1) having undergone MRI imaging at other hospitals, and (2) pre-existing diagnosis of prostate cancer before the prostate biopsy. Patient allocation to the MRI-directed fusion biopsy (MFB) or systematic biopsy (SB) group was primarily determined according to physician preference, patient preference, and clinical decision-making. The study was conducted in accordance with the ethical principles outlined in the Declaration of Helsinki (2013). Informed consent was waived by the Institutional Review Board and Ethics Committee of Shin Kong Wu Ho-Su Memorial Hospital due to the retrospective nature of the study (Waiver approval number: 20250311R).

### 2.2. Operation Procedure

Patients were divided into two groups: the MRI-directed fusion biopsy (MFB) group and the systematic biopsy (SB) group. In the MFB group, patients underwent mpMRI using a 3 Tesla MRI machine (Philips Healthcare, Best, The Netherlands). A qualified radiologist analyzed the MRI images, assessing Prostate Imaging-Reporting and Data System (PI-RADS) scores ranging from 3 to 5. Under heavy sedation, patients were positioned in lithotomy. Utilizing the Urostation system (Koelis, Grenoble, France), a fusion of MRI and real-time ultrasound imaging was employed. Three to six cores of transperineal prostate biopsy were performed based on the size of the identified lesion using this navigation system. Additionally, all patients underwent a random systemic transperineal prostate biopsy consisting of 12 cores. In the SB group, patients were positioned in lithotomy under intravenous general anesthesia, and a random 12-core transrectal prostate biopsy was conducted, guided by real-time transrectal ultrasonography. In the MFB cohort, patients underwent combined targeted transperineal biopsy and systematic transperineal biopsy, and patients in the SB cohort underwent conventional 12-core systematic transrectal biopsy. Therefore, the present study compared two different diagnostic strategies rather than isolated MRI-targeted biopsy alone.

### 2.3. Assessment of Study Interests

A retrospective analysis of clinical and pathological data was conducted by reviewing medical records. Clinical data included age, PSA levels, and prostate size, and pathological data were recorded. Clinically significant PCa (csPCa) was defined as a Gleason score ≥ 7 and high-grade PCa was defined as Gleason score ≥ 8.

### 2.4. Statistical Analysis

Continuous variables were expressed as mean ± standard deviation (SD), and categorical variables were expressed as numbers (percentage). Statistical significance for continuous variables was determined using Student’s *t*-tests, while categorical variables were compared utilizing Chi-square tests or Fisher’s exact tests. Continuous variables were visually inspected for approximate normal distribution before parametric testing. Additional complete-case multivariable logistic regression analyses were performed to evaluate independent associations between biopsy strategy and detection of PCa, csPCa, and high-grade PCa after adjustment for age, PSA level, and PSA density. Odds ratios (ORs) with 95% confidence intervals (CIs) were calculated. Because MRI findings were available only in the MFB cohort, a separate subgroup multivariable logistic regression analysis was performed to evaluate the independent association between PI-RADS category and cancer detection outcomes. In this model, PI-RADS category, age, PSA level, and PSA density were included as covariates. Sensitivity analyses were performed using complete-case data to assess the robustness of the findings. All statistical analyses were performed using SPSS version 22.0 (IBM, Armonk, NY, USA). Two-sided *p* values were calculated, with a level of <0.05 considered statistically significant.

## 3. Results

Initially, 283 patients with PSA levels ranging between 4–20 ng/mL and no palpable prostate nodules were enrolled. Subsequently, 21 patients were excluded from the study, comprising 20 individuals who had undergone MRI imaging at other hospitals, and one patient with a pre-existing diagnosis of prostate cancer prior to the prostate biopsy. As a result, the final analysis included a total of 262 patients, with 89 patients in the MFB group and 173 patients in the SB group.

The patient characteristics, including age, PSA levels, and prostate size, were summarized in Table 1. The mean age was 64.6 ± 8.3 in the MFB group and 68.1 ± 8.3 in the SB group (*p* = 0.0028). The PSA levels were 9.7 ± 3.6 in the MFB group and 9.4 ± 4.1 in the SB group (*p* = 0.087). The prostate volume was 46.9 ± 21.7 in the MFB group and 48.1 ± 21.9 in the SB group (*p* = 0.82). No statistically significant differences were observed between the two groups.

The comparison of prostate cancer detection outcomes between the MFB and SB groups is presented in Table 2. MFB demonstrated significantly higher detection rates of overall PCa, clinically significant PCa, and high-grade PCa compared with SB. Patients in the MFB group were stratified into three groups based on the PI-RADS score: 23 patients (25.8%) in PI-RADS 3, 46 patients (51.7%) in PI-RADS 4, and 20 patients (22.8%) in PI-RADS 5. The subgroup analysis is presented in Table 3. Patients in PI-RADS 5 demonstrated a significantly higher association with PCa compared to those in PI-RADS 3 or PI-RADS 4 (*p* ≤ 0.001). Regarding csPCa, patients in PI-RADS 5 exhibited a higher detection rate compared to the other groups (*p* ≤ 0.001). Furthermore, patients in PI-RADS 5 showed a notably higher rate of high-grade PCa detection compared to other PI-RADS groups (*p* = 0.015).

In a complete-case sensitivity analysis using the available raw data with complete age, PSA, PSA density, and cancer outcome variables (MFB *n* = 89; SB *n* = 173), the MFB strategy remained independently associated with overall PCa detection after an adjustment for age, PSA level, and PSA density. The PSA density was also independently associated with overall PCa detection (adjusted OR 1.54, 95% CI 1.27–1.87, *p* < 0.001). Age remained a significant predictor, whereas PSA level alone was not independently associated with cancer detection (Table 4). Similar findings were observed for clinically significant and high-grade prostate cancer. The MFB strategy remained independently associated with csPCa detection (adjusted OR 2.83, 95% CI 1.58–5.08, *p* < 0.001) and high-grade PCa detection (adjusted OR 6.15, 95% CI 2.49–15.19, *p* < 0.001). The PSA density also demonstrated independent associations with csPCa (adjusted OR 1.67, 95% CI 1.33–2.10, *p* < 0.001) and high-grade PCa (adjusted OR 1.72, 95% CI 1.25–2.36, *p* = 0.001).

In addition, the subgroup multivariable analysis confirmed that MRI findings independently contributed to cancer detection (Table 5). Patients with PI-RADS 5 lesions demonstrated substantially higher odds of overall PCa, csPCa, and high-grade PCa detection compared with patients with PI-RADS 3 lesions. These findings provide formal statistical evidence supporting the diagnostic value of PI-RADS classification and address the incremental contribution of MRI beyond conventional clinical parameters. Taken together, our results support contemporary risk-adapted diagnostic pathways that integrate MRI findings with PSA density rather than relying on either variable alone.

## 4. Discussion

In this retrospective cohort study, MFB was associated with higher detection rates of PCa compared with SB (45.0% vs. 31.8%, *p* = 0.036). This heightened cancer detection rate was particularly prominent in cases of csPCa (40.4% vs. 22.5%, *p* = 0.002) and high-grade PCa (20.2% vs. 5.8%, *p* ≤ 0.001) within the compared groups. Additionally, a strong correlation was observed between the PI-RADS score and the presence of PCa, csPCa, and high-grade PCa (*p* < 0.001, *p* < 0.001, and *p* = 0.015, respectively). To the best of our knowledge, this study represents the first investigation in Taiwan comparing the PCa detection rates between MFB and SB. However, these findings should be interpreted cautiously because patient allocation was not randomized and the MFB cohort underwent a combined targeted and systematic transperineal biopsy, whereas the SB cohort underwent a systematic transrectal biopsy alone. The improved cancer detection observed in the MFB group may not solely reflect MRI targeting. The combination of targeted biopsy, additional systematic sampling, and the transperineal approach may collectively contribute to an improved detection and grading performance [10].

In the PRECISION trial, a multicenter, randomized, non-inferiority study, 500 men were enrolled and randomly assigned [6]. These men, suspected of having PCa, was randomly allocated to undergo MRI, with or without targeted biopsy, or standard TRUSP-Bx. The MRI-targeted biopsy group revealed csPCa in 38% of cases, in contrast to 26% in the TRUSP-Bx group (*p* = 0.005). Notably, 71 out of 252 men (28%) were spared from undergoing a biopsy due to negative MRI findings. In the PROMIS trial, using mpMRI to screen men allowed 27% of patients to avoid a biopsy. Moreover, the MRI-Targeted biopsy approach detected 18% more cases of csPCa compared to the standard systematic biopsy approach [1]. Another meta-analysis revealed that patients with a positive MRI showed a considerably higher detection rate of Gleason score ≥ 7 cancers through an MRI-targeted biopsy compared to a systematic biopsy (in prospective cohort studies, *p* < 0.001; in retrospective cohort studies, *p* = 0.004) [11]. mpMRI images not only improved the detection rate of csPCa but also helped avoid unnecessary biopsies.

The incidence of Gleason score 6 patients detected via an MRI-targeted biopsy was notably lower compared to a systematic biopsy (14% vs. 25%, *p* < 0.001) [12]. Another study revealed that an MRI-targeted biopsy detected a smaller percentage of patients with clinically insignificant PCa than a systematic biopsy (5.6% vs. 19.5%, *p* < 0.0001) [13]. Notably, an MRI-targeted biopsy, when conducted without a systematic biopsy, markedly reduced the overdiagnosis of low-risk disease in contrast to the systematic biopsy [14]. Conversely, our research incorporated a systemic transperineal prostate biopsy routinely for patients in the MFB group to ensure comprehensive cancer detection. Moreover, there were no instances where a positive finding occurred on the systematic biopsy but not on the MRI-targeted biopsy in our study.

MRI exhibits a high sensitivity in detecting and localizing of Gleason score ≥ 7 cancers, particularly when their size exceeds 10 mm [15]. A Cochrane meta-analysis revealed an aggregated sensitivity of 0.91 and specificity of 0.37 for identifying Gleason score ≥ 7 cancers [7]. However, MRI’s sensitivity is comparatively lower in detecting Gleason score = 6 PCa, capturing less than 30% of those smaller than 0.5 cc as identified through a histopathology analysis of radical prostatectomy specimens [16]. A meta-analysis of 17 studies indicates that lesions with PI-RADS scores of 3, 4, and 5 had average positive predictive values for Gleason score ≥ 7 cancers at 16% (ranging from 7% to 27%), 59% (ranging from 39% to 78%), and 85% (ranging from 73% to 94%), respectively [17]. However, significant heterogeneity existed among these studies. In comparison to other works of research, our study found positive predictive values for Gleason score ≥ 7 cancers of lesions with PI-RADS scores of 3, 4, and 5 at 13%, 37%, and 80%, respectively. Notably, our study’s positive predictive values were not inferior to those reported in other studies.

While our study did not observe severe complications, it highlighted an additional advantage of the transperineal approach: notably lower infection rates compared to TRUSP-Bx. In the PREVENT randomized trial, 658 participants were randomly assigned to undergo either a transrectal or transperineal biopsy, which demonstrated a lower infection rate in the transperineal group (1.6% in the transrectal group vs. 0% in the transperineal group) with a similar cancer diagnosis rate [18]. A comprehensive meta-analysis indicated a significantly reduced risk of infectious complications with the transperineal biopsy in contrast to the transrectal biopsy (Relative Risk: 0.55) [19]. Moreover, a systematic review including 162,577 patients demonstrated notably lower sepsis rates of 0.1% for transperineal biopsies compared to 0.9% for transrectal biopsies [20]. Another study conducted in the United Kingdom revealed decreased re-admission rates for sepsis in patients who underwent transperineal biopsies compared to transrectal biopsies (1.0% vs. 1.4%) [21]. Accumulating evidence supports the broader adoption of the transperineal approach because of its lower infectious complication rate.

Based on previous systematic reviews and meta-analyses evaluating the relationship between PI-RADS classification and prostate cancer detection rates, Oerther et al. reported that the cancer detection rate for PI-RADS 3 lesions ranges from 43% to 61% [17]. However, subsequent investigations have demonstrated substantially lower detection rates, typically ranging from 13% to 20% [22,23]. A further review by Satei et al. reported an overall prostate cancer incidence of 21.4% in PI-RADS 3 lesions, with only a subset representing clinically significant prostate cancer (csPCa), defined as a Gleason score ≥ 7 [24]. These findings highlight the considerable heterogeneity in reported detection rates and underscore the uncertainty surrounding the clinical significance of PI-RADS 3 lesions. In our study, the cancer detection rate for PI-RADS 3 lesions was 13%, which is consistent with the lower range reported in the literature. This relatively low predictive value suggests that the routine biopsy of all PI-RADS 3 lesions may lead to overdiagnosis, unnecessary invasive procedures, and increased patient morbidity without substantial clinical benefit.

Given these limitations, recent studies have increasingly emphasized the importance of integrating additional clinical and radiological parameters to refine biopsy decision-making. For instance, Franz et al. conducted a large retrospective cohort study including 671 patients with a total of 981 PI-RADS 3 lesions, demonstrating that clinically significant prostate cancer was detected in only 15.8% of lesions. Their analysis further identified prostate-specific antigen density (PSAD), older age, and higher serum PSA levels as significant predictors of both overall prostate cancer and csPCa. Among these factors, PSAD consistently emerged as the strongest independent predictor, reinforcing its clinical utility in risk stratification [25]. Similarly, Wee et al. retrospectively analyzed 214 transition zone PI-RADS 3 lesions and reported that both clinical and imaging parameters could significantly improve risk stratification. Specifically, clinical variables such as PSAD ≥ 0.11 ng/mL/cm^3^ and Prostate Health Index (PHI) ≥ 34 were strongly associated with an increased likelihood of csPCa. In addition, imaging characteristics—including marked restricted diffusion (DWI score ≥ 4), lesion diameter ≥ 1 cm, and apical lesion location—were also significantly correlated with malignancy. These findings suggest that combining quantitative biomarkers with detailed MRI features may enhance the predictive accuracy beyond PI-RADS classification alone [26]. Furthermore, a large multi-institutional analysis by Fang et al. [27], which included 1784 patients with PI-RADS 3 lesions, demonstrated that a higher PSAD, advanced age, and biopsy-naïve status were independently associated with the presence of clinically significant prostate cancer. Notably, patients with a history of a prior negative biopsy exhibited a significantly lower risk of cancer detection, indicating that prior biopsy status should also be considered when determining the necessity of a repeat biopsy. These findings further support the concept that PI-RADS 3 lesions should not be managed uniformly, but rather through an individualized risk assessment.

Across these studies, PSAD has consistently been identified as one of the most reliable and reproducible predictors of prostate cancer in PI-RADS 3 lesions. Supporting this observation, Drevik et al. [23] conducted a multi-institutional study involving 349 patients and reported that a PSAD threshold greater than 0.15 ng/mL/cm^3^ was significantly associated with increased cancer detection rates in PI-RADS 3 lesions. This threshold has also been widely adopted in clinical guidelines as a practical cutoff to guide biopsy decisions, particularly in patients with equivocal MRI findings. Taken together, the current evidence indicates that PI-RADS 3 lesions represent a heterogeneous group with relatively low and variable cancer detection rates. The reliance on PI-RADS classification alone is insufficient for clinical decision-making. Instead, a comprehensive, risk-adapted approach that incorporates clinical parameters (such as PSAD, PSA level, age, and biopsy history) alongside imaging features (including lesion size, diffusion characteristics, and anatomical location) is essential to improve diagnostic accuracy. Such an approach may reduce unnecessary biopsies while maintaining the ability to detect clinically significant disease. Nevertheless, further prospective, large-scale studies are warranted to validate optimal risk stratification models and to establish standardized criteria for biopsy decision-making in patients with PI-RADS 3 lesions. An improved understanding of the prognostic factors and the integration of novel biomarkers may further enhance individualized patient management and optimize clinical outcomes.

Magnetic resonance imaging (MRI)-fusion biopsy has been shown to significantly reduce the number of unnecessary prostate biopsies by improving lesion targeting and diagnostic precision. However, this technique is associated with higher procedural and infrastructural costs, which raises important concerns regarding its overall cost-effectiveness. Several studies have therefore focused on evaluating the economic impact of MRI-guided biopsy strategies compared with conventional transrectal ultrasound (TRUS)-guided systematic biopsy. Evidence from economic analyses suggests that, although MRI-guided biopsy incurs higher upfront costs, these may be offset by the improved detection of clinically significant prostate cancer and a reduction in overdiagnosis and overtreatment, ultimately leading to better quality-adjusted life-year (QALY) outcomes [28]. Barnett et al. proposed a risk-adapted strategy in which patients with Prostate Imaging Reporting and Data System (PI-RADS) scores ≥ 3 undergo a combined biopsy (targeted plus systematic biopsy), while biopsies may be safely avoided in patients with PI-RADS scores < 3. This approach aims to optimize diagnostic efficiency and minimize unnecessary invasive procedures, thereby improving cost-effectiveness. Supporting this concept, systematic reviews have demonstrated that MRI-targeted biopsy strategies are generally cost-effective in both initial and repeat biopsy settings, particularly in patients with persistent clinical suspicion or a prior negative biopsy [29]. Additionally, decision analysis models provided by Davuluri et al. indicate that an MRI-fusion biopsy may be more cost-effective than a repeat TRUS biopsy in patients with previous negative results, although it may not always be superior in the initial biopsy setting due to higher costs [30]. In Taiwan, the National Health Insurance system provides broad coverage for fundamental healthcare services, which may facilitate access to prostate cancer diagnostic tools. Nevertheless, the relatively high cost of MRI and fusion biopsy technology remains a barrier to widespread implementation. There is a lack of region-specific economic evaluations addressing the role of MRI-fusion biopsy in Taiwan. Further large-scale, prospective studies incorporating local cost data, healthcare resource utilization, and long-term clinical outcomes are required to validate the cost-effectiveness of MRI-fusion biopsy.

This first investigation in Taiwan found that MFB was associated with a higher detection rate of PCa compared with SB. This heightened cancer detection rate was notably pronounced in identifying csPCa and high-grade PCa within the compared groups. Furthermore, a robust correlation was observed between the PI-RADS score and the presence of PCa, csPCa, and high-grade PCa. Nevertheless, because the study compared two biopsy strategies with different routes and sampling approaches, the results should not be interpreted as proof that MRI targeting alone was responsible for the observed differences. Our study had several distinct advantages. Firstly, all patients underwent mpMRI utilizing a 3 Tesla MRI machine. Secondly, the assessment of the PI-RADS score was consistently performed by the same experienced radiologist. Thirdly, every patient in the MFB group was evaluating using the Urostation system (Koelis, Grenoble, France). We believed that these factors helped minimize procedural variability and strengthened the consistency of our findings.

### 4.1. Clinical Implications

The results also emphasize that PI-RADS 3 lesions should not be managed uniformly. The low PI-RADS 3 detection rate in this cohort supports the integration of hte PSA density, PSA level, patient age, prior biopsy history, lesion size, diffusion restriction, and Tlesion location when deciding whether to biopsy equivocal lesions. Such individualized decision-making may reduce unnecessary biopsies while maintaining the detection of clinically significant disease [17,23,25,26,27].

The present findings support a risk-adapted diagnostic pathway for men with moderately elevated PSA and no palpable prostate nodules. An MRI-directed biopsy may be particularly useful for identifying csPCa and high-grade PCa, which are the outcomes most relevant to treatment decision-making. In clinical practice, this supports the use of mpMRI before a biopsy and the selective targeting of suspicious lesions, consistent with contemporary guideline recommendations that MRI should be performed before a biopsy in men with suspected organ-confined prostate cancer [3].

### 4.2. Limitation

Several limitations should be acknowledged. Firstly, the PI-RADS score underwent an evaluation by a single radiologist without subsequent verification. However, despite this limitation, our study demonstrated a similar cancer detection rate in correlating the PI-RADS score with PCa compared to other sources. Secondly, our investigation solely focused on comparing the PCa detection rates between groups, lacking comprehensive data on cancer-specific survival or overall survival within a short follow-up period. Thirdly, in cases where a negative finding was observed in the TRUSP-BX group, there existed a potential for the missed detection of cancerous lesions. In such instances, our protocol involved a regular PSA follow-up initially, followed by further management if necessary.

Fourthly, because patient assignments were not randomized, the possibility of selection bias should be acknowledged. Patients who underwent mpMRI before a biopsy may have represented a subgroup with greater clinical suspicion for prostate cancer. The present study did not include a formal prospective sample size calculation because of its retrospective design. Therefore, the possibility of insufficient statistical power for certain subgroup analyses should be acknowledged. Although multivariable logistic regression analyses were performed, incorporating age, PSA level, and PSA density, important variables including family history, comorbidity burden, prior biopsy status, and additional MRI characteristics were not consistently available and therefore could not be included in the final regression models. Furthermore, MRI findings were available only in the MFB cohort, requiring separate subgroup analyses rather than inclusion within the primary regression model. Consequently, residual confounding cannot be excluded, and the observed associations between the biopsy strategy and cancer detection outcomes should be interpreted with caution. Future prospective studies with comprehensive covariate collection and propensity-score or matched analyses are warranted to validate these findings. A further limitation of the present study is the inability to perform adequately powered subgroup analyses according to the PSA level or prostate volume because of the relatively limited sample size, particularly within the MFB cohort. Additional stratification substantially reduced the subgroup numbers and may have resulted in unstable statistical estimates. Larger prospective studies are therefore needed to validate the consistency of these findings across different clinical subgroups.

## 5. Conclusions

In this retrospective cohort study, an MRI-directed fusion biopsy combined with a systematic transperineal biopsy was associated with higher detection rates of PCa, csPCa, and high-grade PCa compared with a conventional systematic transrectal biopsy. The PSA density and MRI findings were independently associated with cancer detection outcomes, suggesting that a combined clinical and imaging risk stratification may improve patient selection for biopsy. Because of the retrospective design and potential residual confounding, these findings should be interpreted as associations rather than evidence of causality. Further prospective studies are warranted to validate these observations.

## Figures and Tables

**Table 1:** Baseline demographic and clinical characteristics of patients undergoing MRI-directed fusion biopsy (MFB) or conventional systematic biopsy (SB). Continuous variables are presented as mean ± standard deviation. PSA = prostate-specific antigen. *p*-values were calculated using Student’s *t*-test.

	Fusion Biopsy	Systematic Biopsy	*p*-Value
Number	89	173	
Age, years	64.6 ± 8.3	68.1 ± 8.3	0.0028
PSA, ng/mL	9.7 ± 3.6	9.4 ± 4.1	0.087
Prostate volume, mL	46.9 ± 21.7	48.1 ± 21.9	0.820

**Table 2:** Comparison of prostate cancer detection rates between MRI-directed fusion biopsy (MFB) and conventional systematic biopsy (SB). Data are presented as number (%). Clinically significant prostate cancer (csPCa) was defined as Gleason score ≥ 7, and high-grade prostate cancer was defined as Gleason score ≥ 8. *p*-values were calculated using Chi-square or Fisher’s exact tests.

	Fusion Biopsy	Systematic Biopsy	*p*-Value
Number	89	173	
PCa	40 (45.0%)	55 (31.8%)	0.036
Clinically significant PCa	36 (40.4%)	39 (22.5%)	0.002
High-grade PCa	18 (20.2%)	10 (5.8%)	<0.001

**Table 3:** Correlation between PI-RADS score and prostate cancer detection rates in the MRI-directed fusion biopsy cohort. Data are presented as number (%). Clinically significant prostate cancer (csPCa) was defined as Gleason score ≥ 7. PI-RADS = Prostate Imaging Reporting and Data System. *p*-values were calculated using Chi-square or Fisher’s exact tests.

	PI-RADS 3	P-RADS 4	PI-RADS 5	*p*-Value
Number	23	46	20	
PCa	3 (13.0%)	21 (45.7%)	16 (80.0%)	<0.001
Clinically significant PCa	3 (13.0%)	17 (37.0%)	16 (80.0%)	<0.001
High-grade PCa	1 (4.3%)	9 (19.6%)	8 (40.0%)	0.015

**Table 4:** Complete-case multivariable logistic regression analysis for cancer detection outcomes.

Outcome	Adjusted Predictor	Adjusted OR	95% CI	*p* Value
Overall PCa	MFB strategy	2.01	1.16–3.48	0.013
Overall PCa	Age	1.04	1.01–1.08	0.010
Overall PCa	PSA	1.05	0.98–1.12	0.149
Overall PCa	PSA density	1.54	1.27–1.87	<0.001
csPCa	MFB strategy	2.83	1.58–5.08	<0.001
csPCa	PSA density	1.67	1.33–2.10	<0.001
High-grade PCa	MFB strategy	6.15	2.49–15.19	<0.001
High-grade PCa	PSA density	1.72	1.25–2.36	0.001

**Table 5:** Multivariable logistic regression analysis of MRI findings within the MFB cohort.

Outcome	Adjusted Predictor	Adjusted OR	95% CI	*p* Value
PCa	PI-RADS 4 vs. 3	2.14	1.05–4.36	0.035
PCa	PI-RADS 5 vs. 3	7.38	2.18–24.98	0.001
csPCa	PI-RADS 5 vs. 3	8.42	2.49–28.49	<0.001
High-grade PCa	PI-RADS 5 vs. 3	6.93	1.82–26.41	0.005

## Data Availability

The data presented in this study are available upon reasonable request from the corresponding author. The data are not publicly available due to privacy and ethical restrictions.

## Abbreviations

The following abbreviations are used in this manuscript:

PSA	prostate-specific antigen
PCa	prostate cancer
csPCa	clinically significant prostate cancer
TRUSP-Bx	transrectal ultrasonography–guided biopsy
MFB	MRI-directed fusion biopsy
PI-RADS	Prostate Imaging–Reporting and Data System
